# Pre-hospital pain management; a systematic review of proposed guidelines 

**Published:** 2019-10-06

**Authors:** Mahmoud Yousefifard, Shaghayegh Askarian-Amiri, Arian Madani Neishaboori, Mostafa Sadeghi, Peyman Saberian, Alireza Baratloo

**Affiliations:** 1 Prevention of Cardiovascular Disease Research Center, Shahid Beheshti University of Medical Sciences, Tehran, Iran.; 2 Physiology Research Center, Faculty of Medicine, Iran University of Medical Sciences, Tehran, Iran.; 3 Department of Anesthesiology and Critical Care, Shariati Hospital, Tehran University of Medical Sciences, Tehran, Iran.; 4 Department of Anesthesiology, Imam Khomeini Hospital Complex, Tehran university of Medical Sciences, Tehran, Iran.; 5 Prehospital and Hospital Emergency Research Center, Tehran University of Medical Sciences, Tehran, Iran.; 6 Department of Emergency Medicine, Sina Hospital, Tehran University of Medical Sciences, Tehran, Iran.

**Keywords:** Pain management, practice guideline, drug therapy, Emergency Medical Services

## Abstract

**Introduction:**

A standard guideline concerning pre-hospital pain management is still a matter of discussion. Therefore, the current umbrella review is determined to perform a comprehensive search in databases and Grey literature and collect and summarize the guidelines and protocols dealing with prehospital pain management.

**Methods:**

In the present study, all of the available guidelines and protocols concerning pre-hospital pain management were reviewed. Presented guidelines are from 2010 up to present, as the majority of guidelines are considered old and become renewed after 10 years. Finally, the development quality of each guideline was evaluated using AGREE II instrument.

**Results:**

The search conducted in databases and non-indexed protocols resulted in inclusion of 12 pre-hospital pain management guidelines. The time interval of the guidelines was from 2010 to 2019. Four guidelines were designed for pain management in trauma patients and other guidelines were presented for all of the clinical conditions associated with pain. All of the 12 included guidelines presented pain management instructions in adults. Pain management in children was reported in 10 guidelines. All of the guidelines persisted on a standard method for pain evaluation. Pain management was categorized in three groups; mild, moderate and severe pain. Most of the guidelines recommend paracetamol as an optional treatment for management of mild pain in both adults and children. In management of moderate and severe pain, fentanyl and morphine were suggested for both adults and children. In most of the treatment guidelines fentanyl is the optional choice for children.

**Conclusion:**

The present umbrella review has summarized the current evidence in pre-hospital pain management for the first time via investigation of guidelines and protocols related to the matter. Based on the obtained evidence, no guideline is yet presented concerning opioid-free management of moderate and severe pain. The evidence is insufficient for using non opioid medications such as ketamine.

## Introduction

Pain management has been a priority for prehospital and hospital care, and a variety of guidelines have been adopted accordingly (1). Existence of moderate to severe pain is one of the most important factors, which alternates patients’ conditions and might have a negative impact on their physiological parameters, which could eventually worsen the patient’s prognosis (2, 3). Pharmacological treatment choices in prehospital pain management are quite limited, considering that in prehospital care, analgesics should not only be effective and safe, but also not interfere with patients’ transfer (1). 

Based on literature reviews done in recent years and different expert panels conducted, several guidelines have been proposed for pain management in prehospital care (4-6). These guidelines suggest different treatment options varying from injectable opioids to intranasal non-opioid treatments. Nevertheless, instructions given by these protocols are usually taken from moderate to low quality studies (7), thus, there is no consensus over a single guideline. On the other hand, the majority of these guidelines only refer to a single disease or complication. In these instructions a broad range of analgesic drugs are proposed. Some of the guidelines suggest using multiple drugs instead of a single medication; and some others suggest using opioids in lower doses (4-8).

Clearly, there is not a consensus over establishing standard instructions on pre-hospital pain control. Hence, the present systematic review aims to collect and summarize pre-hospital pain management guidelines and instructions by conducting an extensive research in databases.

## Method:

**Study design and search strategy**

The current study is an umbrella review, which investigates the guidelines and protocols for pre-hospital pain management. The researchers conducted a comprehensive search in electronic databases. Appropriate keywords were defined to accomplish the study’s objectives. For this purpose, “pre-hospital emergencies” and “analgesia” related words were obtained with advice of an experienced researcher in the field. Additionally, MeSh and Emtree word trees were investigated. The attained keywords were properly combined, and standard tags were adapted for each database. Subsequently, a comprehensive search was conducted in electronic databases including: Medline, Embase, Trip Medical Database and Scopus through March 2019. Search strategy in Medline database is presented in Panel 1. In addition to systematic search, manual search was also performed in Google search engine, Google scholar and references of relevant articles. 

**Selection criteria**

In the present study, guidelines and protocols concerning pre-hospital pain management, published in peer-review journals or released in valid organizations’ websites, were included. Reviews being narrative, lack of a report on the complete process of the guideline’s extraction, and lack of a report on the systematic review’s process were considered as exclusion criteria. 

**Data collection and quality assessment**

The data collected from databases were saved in Endnote. Two independent researchers studied the records and screened titles and abstracts of relevant guidelines. After studying the full text of these guidelines, data were filed in a checklist created in Microsoft Excel. Obtained results were perused by the two researchers with the presence of a third researcher. Any disagreement was discussed and resolved. 

Recorded data in the checklist consisted of name of the guideline, year of publication, studied medications, quality control and patients’ conditions (trauma, etc.). In cases of non-extractable data in the articles, their authors were contacted. If the author did not respond to the first email, a reminder was sent. In case of no response, second reminder email was sent within two weeks. Granted that still no response was received, the other authors were contacted via social media such as ResearchGate and LinkedIn to attain required data.

**Panel 1:** Search query in medline

Search terms
1- “Emergency Medical Services”[mh] OR “Emergency Health Service”[tiab] OR “Emergency Care”[tiab] OR “Prehospital Medication”[tiab] OR “Prehospital Care”[tiab] OR “Prehospital”[tiab] OR “Emergency Services, Medical”[tiab] OR “Emergency Service, Medical”[tiab] OR “Medical Emergency Service”[tiab] OR “Medical Emergency Services”[tiab] OR “Service, Medical Emergency”[tiab] OR “Services, Medical Emergency”[tiab] OR “Medical Services, Emergency”[tiab] OR “Emergency Medical Service”[tiab] OR “Medical Service, Emergency”[tiab] OR “Service, Emergency Medical”[tiab] OR “Services, Emergency Medical”[tiab] OR “Prehospital Emergency Care”[tiab] OR “Emergency Care, Prehospital”[tiab] OR “Emergicenters”[tiab] OR “Emergicenter”[tiab] OR “Emergency Care”[tiab] OR “Emergency Health Services”[tiab] OR “Emergency Health Service”[tiab] OR “Health Service, Emergency”[tiab] OR “Health Services, Emergency”[tiab] OR “Service, Emergency Health”[tiab] OR “Services, Emergency Health”[tiab] 2- Patient Controlled Analgesia[tiab] OR Analgesic Drugs[tiab] OR Drugs, Analgesic[tiab] OR Anodynes[tiab] OR Analgesic Agents[tiab] OR Agents, Analgesic[tiab] OR Analgesics, Non Narcotic[tiab] OR Non-Narcotic Analgesics[tiab] OR Nonopioid Analgesics[tiab] OR Analgesics, Nonopioid[tiab] OR Non-Opioid Analgesics[tiab] OR Analgesics, Non-Opioid[tiab] OR Non Opioid Analgesics[tiab] OR Analgesics, Nonnarcotic[tiab] OR Nonnarcotic Analgesics[tiab] OR Antinociceptive Agents[tiab] OR Opioid Analgesics[tiab] OR Opioids[tiab] OR Partial Opioid Agonists[tiab] OR Agonists, Partial Opioid[tiab] OR Opioid Agonists, Partial[tiab] OR Opioid Partial Agonists[tiab] OR Agonists, Opioid Partial[tiab] OR Partial Agonists, Opioid[tiab] OR Full Opioid Agonists[tiab] OR Agonists, Full Opioid[tiab] OR Opioid Agonists, Full[tiab] OR Opioid Full Agonists[tiab] OR Agonists, Opioid Full[tiab] OR Full Agonists, Opioid[tiab] OR Opioid Mixed Agonist-Antagonists[tiab] OR Agonist-Antagonists, Opioid Mixed[tiab] OR Mixed Agonist-Antagonists, Opioid[tiab] OR Opioid Mixed Agonist Antagonists[tiab] OR Narcotic[tiab] OR Narcotic Analgesics[tiab] OR Analgesics, Narcotic[tiab] OR Narcotic Effect[tiab] OR Effect, Narcotic[tiab] OR Narcotic Effects[tiab] OR Effects, Narcotic[tiab] OR Antiinflammatory Agents, Non Steroidal[tiab] OR NSAIDs[tiab] OR Non-Steroidal Anti-Inflammatory Agents[tiab] OR Non Steroidal Anti Inflammatory Agents[tiab] OR Nonsteroidal Anti-Inflammatory Agents[tiab] OR Nonsteroidal Anti Inflammatory Agents[tiab] OR Anti Inflammatory Agents, Nonsteroidal[tiab] OR Antiinflammatory Agents, Nonsteroidal[tiab] OR Nonsteroidal Antiinflammatory Agents[tiab] OR Analgesics, Anti-Inflammatory[tiab] OR Anti-Inflammatory Analgesics[tiab] OR Aspirin-Like Agents[tiab] OR Aspirin Like Agents[tiab] OR Anesthetic Drugs[tiab] OR Drugs, Anesthetic[tiab] OR Anesthetic Agents[tiab] OR Agents, Anesthetic[tiab] OR Anesthetic Effect[tiab] OR Effect, Anesthetic[tiab] OR Anesthetic Effects[tiab] OR Effects, Anesthetic[tiab] OR Morphinan[tiab] OR “Acetaminophen”[mh] OR “Adapalene”[mh] OR “Adapalene, Benzoyl Peroxide Drug Combination”[mh] OR “Amantadine”[mh] OR “Amitriptyline”[mh] OR “Ampyrone”[mh] OR “Antipyrine”[mh] OR “Apazone”[mh] OR “Aspirin”[mh] OR “Bufexamac”[mh] OR “Carbachol”[mh] OR “Carbamazepine”[mh] OR “Celecoxib”[mh] OR “Clonixin”[mh] OR “Dexmedetomidine”[mh] OR “Diclofenac”[mh] OR “Diflunisal”[mh] OR “Dihydroergotamine”[mh] OR “Dipyrone”[mh] OR “Dronabinol”[mh] OR “Epirizole”[mh] OR “Ergotamine”[mh] OR “Etanercept”[mh] OR “Etodolac”[mh] OR “Etoricoxib”[mh] OR “Fenoprofen”[mh] OR “Feprazone”[mh] OR “Flurbiprofen”[mh] OR “Glafenine”[mh] OR “Ibuprofen”[mh] OR “Indomethacin”[mh] OR “Indoprofen”[mh] OR “Interleukin-2”[mh] OR “Ketoprofen”[mh] OR “Ketorolac”[mh] OR “Ketorolac Tromethamine”[mh] OR “Masoprocol”[mh] OR “Meclofenamic Acid”[mh] OR “Medetomidine”[mh] OR “Mefenamic Acid”[mh] OR “Meloxicam”[mh] OR “Mesalamine”[mh] OR “Methotrimeprazine”[mh] OR “Milnacipran”[mh] OR “Nabumetone”[mh] OR “Naproxen”[mh] OR “Nefopam”[mh] OR “Niflumic Acid”[mh] OR “Nitrous Oxide”[mh] OR “Olopatadine Hydrochloride”[mh] OR “Oxaprozin”[mh] OR “Oxyphenbutazone”[mh] OR “Phenacetin”[mh] OR “Phenylbutazone”[mh] OR “Piroxicam”[mh] OR “Pizotyline”[mh] OR “Quinine”[mh] OR “Resveratrol”[mh] OR “Salicylates”[mh] OR “Sodium Salicylate”[mh] OR “Sulfasalazine”[mh] OR “Sulindac”[mh] OR “Suprofen”[mh] OR “Tolmetin”[mh] OR “Alfentanil”[mh] OR “Alphaprodine”[mh] OR “Buprenorphine”[mh] OR “Buprenorphine, Naloxone Drug Combination”[mh] OR “Butorphanol”[mh] OR “Dextromoramide”[mh] OR “Dextropropoxyphene”[mh] OR “Dihydromorphine”[mh] OR “Diphenoxylate”[mh] OR “Ethylketocyclazocine”[mh] OR “Ethylmorphine”[mh] OR “Etorphine”[mh] OR “Fentanyl”[mh] OR “Hydrocodone”[mh] OR “Hydromorphone”[mh] OR “Levorphanol”[mh] OR “Meperidine”[mh] OR “Meptazinol”[mh] OR “Methadone”[mh] OR “Nalbuphine”[mh] OR “Opiate Alkaloids”[mh] OR “Opium”[mh] OR “Oxycodone”[mh] OR “Oxymorphone”[mh] OR “Pentazocine”[mh] OR “Phenazocine”[mh] OR “Phenoperidine”[mh] OR “Pirinitramide”[mh] OR “Promedol”[mh] OR “Remifentanil”[mh] OR “Sufentanil”[mh] OR “Tapentadol”[mh] OR “Tilidine”[mh] OR “Tramadol”[mh] 3- #1 AND #2

**Table 1:** The characteristics of included guidelines

**Name of guideline**	**Update date**	**Target patients**	**Age group**	**Organization**	**Level of evidence**	**Reference**
Netherlands Association for Emergency Nurses (NAEN) Guideline	2014	Trauma patients	Adults	Netherlands Association for Emergency Nurses	Moderate	(22)
Alabama Department of Public Health EMS (ADPH-EMS) Protocol	2018	Patients with severe pain	Adults; Pediatrics	Alabama State Emergency Medical Control Committee	Moderate	(11)
Douglas County Fire/EMS (Emergency Medical Services)(DCFEMS) Guideline	2017	Trauma patients with severe pain	Adults; Pediatrics	Douglas County Fire/EMS (Emergency Medical Services)	Moderate	(12)
Ambulance Tasmania Clinical Practice (ATCP) Guidelines for Paramedics & Intensive Care Paramedics	2012	General pain management	Adults; Pediatrics	Ambulance Tasmania	High to moderate	(13)
North Carolina College of Emergency Physicians (NCCEP) Protocol	2019	General pain management	Adults; Pediatrics	North Carolina College of Emergency Physicians	High to moderate	(14)
Clinical Practice Guideline of Pre-Hospital Emergency Care Council (PHECC)	2018	General pain management	Adults; Pediatrics	Pre-Hospital Emergency Care Council	Moderate	(15)
Ambulance Victoria Clinical Practice (AVCP) Guideline	2018	General pain management	Adults; Pediatrics	Australia Ambulance Victoria	High to moderate	(16)
Maryland Institute for Emergency Medical Services System (MIEMS) Guideline	2014	General pain management	Adults; Pediatrics	Maryland Institute for Emergency Medical Services System	Moderate	(7)
Italian Intersociety Recommendations on pain management (IIRPM) in the emergency setting	2015	General pain management	Adults; Pediatrics	Italian Intersociety Recommendations on pain management	High to moderate	(18)
New Mexico Pre-hospital Treatment (NMPHT) Guideline	2018	General pain management	Adults; Pediatrics	New Mexico Department of Health	Moderate	(19)
U.S National Association of EMS Physicians (NAEMSP) guideline	2014	Trauma patients	Adults; Pediatrics	U.S NAEMSP Medical Directors Council	High to moderate	(20)
UK National Institute for Health and Care Excellence (NICE) guideline	2016	Trauma patients	Adults	UK National Institute for Health and Care Excellence	High to moderate	(21)

**Table 2:** Recommendations for pre-hospital pain management in adults

**Guideline**	**Pain severity**	**Morphine**	**Fentanyl**	**Ketamine**	**Paracetamol**
**NAEN, 2014**	Moderate	--	1-2 μg/kg every 3 mins (titrate medication on effect)	If hypovolemia or shock state is present 0.25 mg/kg+ Midazolam 1 mg	1000 mg IV for 5 min or 1000 mg oral
	Severe	--	1-2 μg/kg every 3 mins (titrate medication on effect)	If hypovolemia or shock state is present 0.25 mg/kg+ Midazolam 1 mg	1000 mg IV for 5 min titrate until NRS<4
**ADPH-EMS, 2018**	Severe	4 mg initial dose, titrate to pain relief in 2 mg every 3-5 mins, to an initial maximum dose of 10 mg cumulative maximum dose of 25 mg **OR** 0.5 mg IM and cumulative maximum dose of 50 mg	1 μg/kg slow IV/IM/IN to an initial maximum dose of 50 μg. May repeat once.	0.2 mg slow IV to a maximum dose of 25 mg **OR** 0.5 mg IM to a maximum dose of 50 mg	--
**DCFEMS, 2017**	Severe	2-4 mg IV/IO/IM slowly titrate to pain relief to a maximum dose of 10 mg every 10 mins	25 μg IV/IO slowly OR 2 μg/kg IN, titrate to pain relief to a maximum dose of 100 μg every 10 mins	--	--
**ATCP, 2012**	Mild	--	--	--	1000 mg
**ATCP, 2012**	Moderate	Up to 0.05 mg/kg IV/IO (initial maximum dose of maximum 5 mg), titrate to pain relief to a maximum dose of 20 mg every 5 mins	Up to 0.5 μg/kg IV/IO (initial maximum dose of 5 mg), titrate to pain relief to a maximum dose of 200 μg every 5 mins **If the IV access >10 mins delayed/unsuccessful** up to 100 μg IN, titrate to pain relief to a maximum dose of 400 μg every 5 mins	--	1000 mg
	Severe	Up to 0.05 mg/kg IV/IO (initial maximum dose of maximum 5 mg), titrate to pain relief to a maximum dose of 20 mg every 5 mins	Up to 0.5 μg/kg IV/IO (initial maximum dose of maximum 5 mg), titrate to pain relief to a maximum dose of 200 μg every 5 mins	--	--
**NCCEP, 2019**	Mild	--	--	--	15 mg/kg oral
	Moderate to severe	4 mg IV/IO/IM repeat 2 mg every 5 mins if required	50-75 μg IV/IO repeat 25 μg every 20 mins to a maximum 200 μg	--	--
**PHECC, 2018**	Mild	--	--	--	1000 mg oral
	Moderate	--	--	--	1000 mg oral
	Severe	4 mg IV, repeat 2 mg to pain relief to a maximum dose of 16 mg every 2 mins	100 μg IN or 50 μg IV, repeat IN once only after 10 min if needed	0.1 mg/kg IV, repeat once only after 10 min if needed	1000 mg IV
**AVCP, 2018**	Mild	--	--	--	1000 mg oral; 500 mg if weight < 60 kg or frail or elderly, malnourished or liver disease
	Moderate	Up to 5 mg IV, titrate to pain relief every 5 mins (consult after 20 mg)	Up to 50 μg IV, titrate to pain relief every 5 mins (consult after 200 μg) **OR** 200 μg IN repeat up to 50 μg IN every 5 minutes (consult after 400 μg)	--	--
	Severe	Up to 5 mg IV, titrate to pain relief every 5 mins (consult after 20 mg) **OR** 10 mg IM, repeat 5 mg IM after 15 minutes once only if required	Up to 50 μg IV, titrate to pain relief every 5 mins (consult after 200 μg) **OR** 200 μg IN repeat up to 50 μg IN every 5 minutes (consult after 400 μg)	Extreme traumatic pain persists to opioid: 10-20 mg IV at 5-10 min intervals; For severe pain 20-30 mg IV at 2 minute interval	--
**MIEMS, 2014**	Moderate to severe	0.1 mg/kg IV/IO, repeat 0.05 mg/kg IV/IO to pain relief every 5 mins	1 μg/kg IV/IO, repeat 0.5 mg/kg IV/IO to pain relief every 5 mins	--	--
**IIRPM, 2015**	Mild	--	--	--	1000 mg
	Moderate	--	--	--	1000 mg
	Severe	4-6 mg IV; 2-3 mg for patients aged >65 years and/or unstable patients	50-100 μg IV	--	--
**NMPHT, 2018**	Moderate to severe	4-10 mg slow IV/IO, titrating 2-4 mg every 10 mins with a maximum dose of 10 mg	25-100 μg IV/IO	--	--
**NAEMSP, 2014**	Moderate	0.1 mg/kg IM to a maximum initial dose of 15 mg	1 μg/kg IN/IM to a maximum dose of 100 μg	0.5 mg/kg IN to a maximum initial dose of 25 mg and maximum cumulative dose of 100 mg	15 mg/kg oral to a maximum dose of 1000 mg
	Severe	0.1 mg/kg IV/IO to a maximum dose of 10 mg	1 μg/kg IV/IO to a maximum dose of 100 μg	0.25 mg/kg IM/IV/IO to a maximum initial dose of 25 mg and maximum cumulative dose of 100 mg	--
**NICE, 2016**	Moderate to severe	Yes (IV first line; dosage not reported)	--	Yes Second line IN	--

**Table 2:** Recommendations for pre-hospital pain management in adults (continue…)

** Guideline**	**Pain severity**	**Midazolam**	**Nitric oxide**	**Ketorolac**	**Ibuprofen**	**Methoxyﬂurane**	**NSAIDs**
**NAEN, 2014**	Moderate	--	--	--	--	--	--
	Severe	--	--	--	--	--	--
**ADPH-EMS, 2018**	Severe	--	Until pain control	--	--	--	--
**DCFEMS, 2017**	Severe	--	--	--	--	--	--
**ATCP, 2012**	Mild	--	--	--	--	--	--
	Moderate	--	--	--	--	--	--
	Severe	--	--	--	--	3 ml (repeat 3 ml if required (maximum 6 ml)	--
**NCCEP, 2019**	Mild	--	--	--	--	--	--
	Moderate to severe	--	--	--	10 mg/kg oral	--	--
**PHECC, 2018**	Mild	--	50:50 mix	30 mg IV/IO OR 60 mg IM (Maximum 60 mg)	--	--	--
	Moderate	--	--	--	400 mg oral	--	--
	Severe	--	50:50 mix	--	600 mg oral	3 ml, repeat once only if needed	--
**AVCP, 2018**	Mild	--	--	--	--	--	--
	Moderate	--	--	--	--	--	--
	Severe	--	--	--	--	3 ml (repeat 3 ml if required (maximum 6 ml)	--
**MIEMS, 2014**	Moderate to severe	--	--	--	--	--	--
**IIRPM, 2015**	Mild	--	--	--	--	--	--
	Moderate	--	--	--	--	--	Yes (dosage not reported)
**IIRPM, 2015**	Severe	--	--	--	--	--	Yes (dosage not reported)
**NMPHT, 2018**	Moderate to severe	--	--	--	--	--	--
**NAEMSP, 2014**	Moderate	--	--	--	--	--	Contraindication in trauma
	Severe	--	Yes (dosage not reported)	30 mg IM once only	10 mg/kg oral to a maximum dose of 800 mg	--	--
**NICE, 2016**	Moderate to severe	--	--	15 mg IV once only	--	--	--

**Table 2:** Recommendations for pre-hospital pain management in adults (continue…)

**Guideline**	**Pain severity**	**Codeine**	**Tramadol**	**hydromorphone**	**diamorphine**	**Aspirin**
**NAEN, 2014**	Moderate	--	--	--	--	--
	Severe	--	--	--	--	--
**ADPH-EMS, 2018**	Severe	--	--	--	--	--
**DCFEMS, 2017**	Severe	--	--	--	--	--
**ATCP, 2012**	Mild	--	--	--	--	--
	Moderate	--	--	--	--	--
	Severe	--	--	--	--	--
**NCCEP, 2019**	Mild	--	--	--	--	--
	Moderate to severe	--	--	--	--	324-650 mg oral
**PHECC, 2018**	Mild	--	--	--	--	--
	Moderate	--	--	--	--	--
	Severe	--	--	--	--	--
**AVCP, 2018**	Mild	--	--	--	--	--
	Moderate	--	--	--	--	--
	Severe	--	--	--	--	--
**MIEMS, 2014**	Moderate to severe	--	--	--	--	--
**IIRPM, 2015**	Mild	--	--	--	--	--
	Moderate	--	--	--	--	--
**IIRPM, 2015**	Severe	30 mg oral	37.5 mg oral	--	--	--
**NMPHT, 2018**	Moderate to severe	--	--	--	--	--
**NAEMSP guideline, 2014**	Moderate	--	--	--	--	--
	Severe	--	--	--	--	--
**NICE, 2016**	Moderate to severe	--	--	--	Yes (dosage not reported)	--
	Mild to moderate	--	--	--	--	--

**Table 3:** Recommendations for pre-hospital pain management in children

**Guideline**	**Pain severity**	**Morphine**	**Fentanyl**	**Ketamine**	**Paracetamol**
**ADPH-EMS, 2018**	Severe	0.1 mg slow IV to a maximum dose of 5 mg	1 μg/kg slow IV/IN to a maximum dose of 50 μg	0.2 mg slow IV to a maximum dose of 25 mg **OR** 0.5 mg IM to maximum dose of 50 mg **OR** 1 mg/kg IN to a maximum dose of 50 mg	--
**DCFEMS, 2017**	Severe	--	1-2 μg/kg IV/IO slowly or 1-2 μg/kg IN Titrate to pain relief to a maximum dose of 100 μg every 10 mins	--	--
**ATCP, 2012**	Mild	--	--	--	15 mg/kg oral
	Moderate	--	25 μg IN for small child (10-24 kg) 50 μg for large child (>=25 kg) Titrate initial IN dose to pain relief (maximum 3 doses) every 5 mins	--	15 mg/kg oral
**ATCP, 2012**	Severe	**Last resort if pain not controlled** Up to 0.1 mg/kg IM (maximum dose of 5 mg), single dose only **OR** Up to 0.05 mg/kg IV, titrate to pain relief to a maximum dose of 0.2 mg/kg every 5 mins	25 μg IN for small child (10-24 kg) 50 μg for large child (>=25 kg) Titrate initial IN dose to pain relief (maximum 3 doses) every 5 mins; If pain not controlled up to 0.5 μg/kg IV, repeat a single dose to pain relief to a maximum dose of 2 μg/kg after 5 min	--	--
**NCCEP, 2019**	Moderate to severe	0.1 mg/kg IV/IO/IM repeat 0.1 mg/kg every 5 mins (maximum 10 mg)	1 μg/kg IV/IO/IM/IN repeat 0.5 μg/kg every 5 mins (maximum 2 μg/kg)	--	--
**PHECC, 2018**	Mild	--	--	--	20 mg/kg oral
	Moderate	--	--	--	20 mg/kg oral
	Severe	0.3 mg/kg PO, for age>=1 year old, repeat 0.1 mg/kg to pain relief to a maximum dose of 0.1 mg/kg IV every 2 mins	1.5 μg/kg IN, for age>=1 year old, repeat IN once only after 10 min if needed	0.1 mg/kg IV, repeat once only after 10 min if needed	If age<=1 years: 7.5 mg/kg IV If age>1 years 15 mg/kg IV
**AVCP, 2018**	Mild	--	--	--	15 mg/kg oral
	Moderate	--	25 μg IN for small child (10-24 kg) 25 to 50 μg IN for medium child (18 to 39 kg) Repeat 3 doses if needed (consult after 3 doses)	--	15 mg/kg oral
**AVCP, 2018**	Severe	0.05 to 0.1 mg/kg IV, titrate to pain relief to a maximum dose of 0.2 mg/kg every 5-10 mins	25 μg IN for small child (10-24 kg) 25 to 50 mg IN for medium child (18 to 39 kg) Repeat 3 doses if needed (consult after 3 doses)	**Extreme traumatic pain persists despite opioid prescription** 0.25 mg/kg IV at 5-10 min intervals (maximum 0.5 mg/kg)	--
**MIEMS, 2014**	Moderate to severe	0.1 mg/kg IV/IO, repeat 0.05 mg/kg IV/IO to pain relief every 5 mins	1 μg/kg IV/IO, repeat 0.5 mg/kg IV/IO to pain relief every 5 mins	--	--
**IIRPM, 2015**	Mild	--	--	--	10-15 mg/kg oral
	Moderate	--	--	--	15 mg/kg IV
	Severe	0.05-0.1 mg/kg	1-2 μg/kg	--	--
**NMPHT, 2018**	Moderate to severe	0.05 mg/kg IV/IO/IM	0.5 to 1 μg/kg IV/IO	--	--
**NAEMSP, 2014**	Moderate	0.1 mg/kg IM to a maximum initial dose of 15 mg	1 μg/kg IN/IM to a maximum dose of 100 μg	0.5 mg/kg IN to a maximum initial dose of 25 mg and maximum cumulative dose of 100 mg	15 mg/kg oral to a maximum dose of 1000 mg
	Severe	0.1 mg/kg IV/IO to a maximum dose of 10 mg	--	0.25 mg/kg IM/IV/IO to a maximum initial dose of 25 mg and maximum cumulative dose of 100 mg	--

**Table 3:** Recommendations for pre-hospital pain management in children (continue…)

**Guideline**	**Pain severity**	**Midazolam**	**Nitric oxide**	**Ketorolac**	**Ibuprofen**	**Methoxyﬂurane**	**NSAIDs**
ADPH-EMS, 2018	Severe	--	Consult with online medical director	--	--	--	--
DCFEMS, 2017	Severe	--	--	--	--	--	--
	Severe	--	--	--	--	--	--
ATCP, 2012	Mild	--	--	--	--	--	--
	Moderate	--	--	--	--	3 ml (repeat 3 ml if required (maximum 6 ml)	--
	Severe	--	--	--	--	3 ml (repeat 3 ml if required (maximum 6 ml)	--
NCCEP, 2019	Moderate to severe	--	--	0.5 mg/kg IV/IO/IM (Maximum 30 mg)	--	--	--
PHECC, 2018	Mild	--	--	--	10 mg/kg oral	--	--
	Moderate	--	50:50 mix	--	10 mg/kg oral	3 ml, for age>=5 years old repeat once only if needed	--
	Severe	--	--	--	--	--	--
AVCP, 2018	Mild	--	--	--	--	--	--
	Moderate	--	--	--	--	3 ml (repeat 3 ml if required (maximum 6 ml)	--
	Severe	--	--	--	--	3 ml (repeat 3 ml if required (maximum 6 ml)	--
MIEMS, 2014	Moderate to severe	--	--	--	--	--	--
IIRPM, 2015	Mild	--	--	--	4-10 mg/kg oral	--	--
	Moderate	--	--	--	--	--	--
	Severe	--	--	--	--	--	Contraindication in trauma
NMPHT, 2018	Moderate to severe	--	--	--	--	--	--
NAEMSP, 2014	Moderate	--	Yes (dosage not reported)	1 mg IM to a maximum dose of 30 mg	10 mg/kg oral to a maximum dose of 800 mg	--	--
	Severe	--	--	0.5 mg/kg IV with a maximum dose of 15 mg	--	--	--

**Table 3:** Recommendations for pre-hospital pain management in children (continue…)

**Guideline**	**Pain severity**	**Codeine**	**Tramadol**	**hydromorphone**	**diamorphine**	**Aspirin**
**ADPH-EMS, 2018**	Severe	--	--	--	--	--
**DCFEMS, 2017**	Severe	--	--	--	--	--
**ATCP, 2012**	Mild	--	--	--	--	--
	Moderate	--	--	--	--	--
	Severe	--	--	--	--	--
**NCCEP, 2019**	Moderate to severe	--	--	--	--	--
**PHECC, 2018**	Mild	--	--	--	--	--
	Moderate	--	--	--	--	--
	Severe	--	--	--	--	--
**AVCP, 2018**	Mild	--	--	--	--	--
	Moderate	--	--	--	--	--
	Severe	--	--	--	--	--
**MIEMS, 2014**	Moderate to severe	--	--	--	--	--
**IIRPM, 2015**	Mild	--	--	--	--	--
	Moderate	1.5 mg/kg oral	1-2 mg/kg	--	--	--
	Severe	--	--	--	--	--
**NMPHT, 2018**	Moderate to severe	--	--	--	--	--
**NAEMSP, 2014**	Moderate	--	--	--	--	--
	Severe	--	--	0.015 mg/kg IM/IV/IO to an initial maximum dose of 2 mg and cumulative maximum dose of 4 mg	--	--

**Table 4:** Quality assessment of prehospital pain management guidelines based on AGREE II recommendation

**Guideline**	**Quality score (%)**						**Overall Quality score**	**Vote to recommend use**		
	**Domain 1**	**Domain 2**	**Domain 3**	**Domain 4**	**Domain 5**	**Domain 6**		**Yes**	**Yes ** **with modification**	**No**
NAEN, 2014	100	100	29	100	96	33	4.5	1	1	0
ADPH-EMS, 2018	100	94	25	100	79	50	6.0	2	0	0
DCFEMS, 2017	100	89	13	100	88	42	4.5	1	1	0
ATCP, 2012	100	100	81	100	92	17	6.5	2	0	0
NCCEP, 2019	100	78	56	100	79	33	5.0	1	1	0
PHECC, 2018	100	100	77	100	100	8	6.0	2	0	0
AVCP, 2018	100	100	75	100	88	25	5.5	2	0	0
MIEMS, 2014	100	100	81	100	92	33	5.0	1	1	0
IIRPM, 2015	100	100	67	100	88	33	4.5	0	1	1
NMPHT, 2018	89	72	13	100	79	42	4.5	1	1	0
NAEMSP, 2014	94	100	83	100	96	33	6.5	2	0	0
NICE, 2016	100	100	81	100	96	33	6.5	2	0	0

Domain 1: Aim and Scope; Domain 2: Stakeholder involvement; Domain 3: Rigor of development; Domain 4: Clarity of presentation; Domain 5: Applicability; Domain 6: Editorial independence.

**Figure 1:**
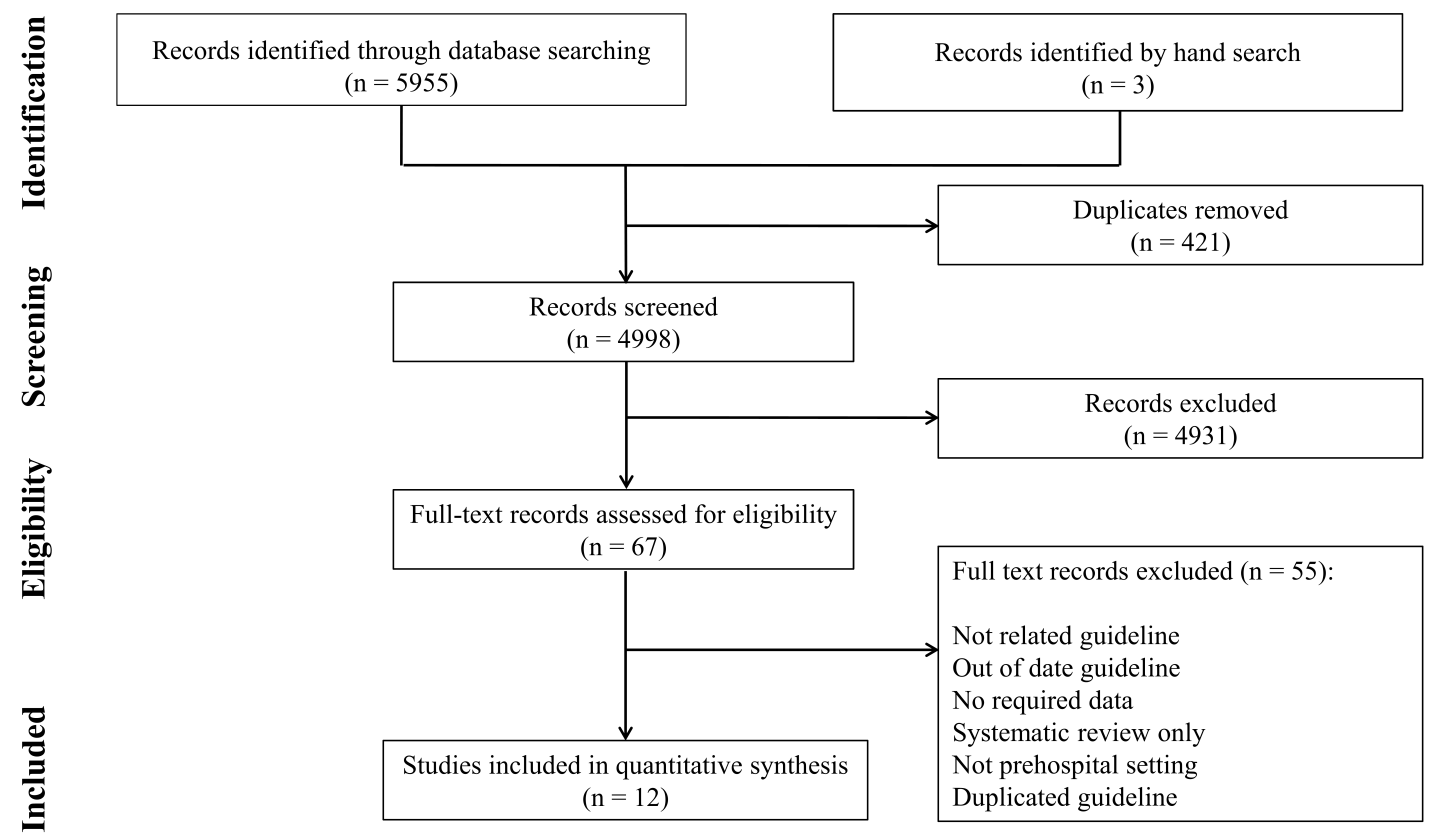
Flow diagram of the present review

**Quality assessment of the articles**

Quality assessment of the articles was performed using AGREE II guideline (9). In order to determine the agreement between the two reviewers, evaluation of Inter-rater reliability in quality assessment of the articles was done. Disagreements were resolved through discussion with a third researcher. 

## Results

**Demographic characteristics of the articles**

Our search in databases and non-indexed guidelines came up with 5988 records. Excluding duplicate records, 4998 articles were found. Reading their titles and abstracts and the full texts of these guidelines, and according to inclusion and exclusion criteria, 12 guidelines for pre-hospital pain management were included in this review (10-21). These guidelines were updated between 2012 and 2019 (Figure 1). Four guidelines were designed for managing pain in trauma patients (10, 12, 20, 21) and the other guidelines were developed for all conditions accompanying pain. All of the 12 included guidelines provided instructions on managing pain in adults, while 10 guidelines (11-20) reported pain management methods in children. Table 1 demonstrates demographic characteristics of these guidelines.

All of the above-mentioned guidelines emphasize on a standard method of pain evaluation. Suggested tools in these guidelines for adults included numeric analog scale (NRS) and visual analog scale (VAS), and for children included The Face, Legs, Activity, Cry, CONSOL ability scale (FLACC) or Children's Hospital of Eastern Ontario Pain Scale (CHEOPS scale), Faces Pain Scale (FPS) scale, FPS-revised, Wong Baker scale and NRS.

**Pain management in adults**

As mentioned previously, pain management in adults was reported in all of the 12 studies. Different medications are suggested in these guidelines which include: fentanyl, morphine, ketamine, paracetamol, midazolam, nitric oxide, ketorolac, ibuprofen, methoxyflurane, nonsteroidal anti-inflammatory drugs, codeine, tramadol and aspirin. Medication protocols are modified in these guidelines based on the severity of pain. 

***Pre-hospital management of mild pain in adults***

Five studies accurately proposed protocols for pre-hospital management of mild pain. Mild pain is described as a severity less than 4, on a 0-10 pain scale. Based on the guidelines included in the present systematic review, management of a patient with mild or endurable pain is prescribing oral paracetamol (1000mg or 15mg/kg). One guideline recommends that if a patient has weighs less than 60 kg, or is older than 60 or is malnourished, the suggested dose for paracetamol should be reduced to half. Only one guideline suggests administration of ketorolac (30mg, IV/IO or 60 mg, IM) instead of paracetamol in relieving mild pain. This guideline proposes administration of nitric oxide in 50:50 dosage as an alternative treatment. 

***Pre-hospital management of moderate pain in adults***

10 guidelines proposed instructions for pre-hospital management of moderate pain. Moderate pain is described as a severity between 4 and 6 on a 0-10 pain scale. Based on the guidelines included in the present systematic review, management of a patient with moderate pain is mainly done by prescribing morphine and fentanyl. However, two guidelines (15, 18) recommend administering paracetamol 1000 mg instead. Using morphine in moderate pain management is mentioned unfavorable in NAEN guideline as well. Rather, it is recommended to use fentanyl (1-2 g/kg) or paracetamol (1000 mg) IV in 5 minutes (or as oral agent). Assuming that the patient is hypovolemic or in shock, this guideline recommends using ketamine (0.25 mg/kg) and midazolam (1 mg) for pain alleviation (10). NICE guideline, recommends using morphine as the first line treatment and ketamine as the second line (21). This guideline does not clarify the dosage and route of administration (Table 2).

Intravenous paracetamol (five guidelines), ketamine (three guidelines), ketorolac (one guideline) and nonsteroidal anti-inflammatory drugs (one guideline) are other recommended options for adults’ pain management in prehospital conditions. Although, one guideline (20) prohibits using nonsteroidal anti-inflammatory drugs in management of Trauma patients (Table 2). 

***Pre-hospital management of severe pain in adults***

12 guidelines proposed instructions on pre-hospital management of severe pain. Severe pain is described as a severity of more than 6 on a 0-10 pain scale. Based on the guidelines included in the present systematic review, prescribing morphine and fentanyl is the first line of treatment in pre-hospital managing severe pain. Nevertheless, guidelines show some controversy. NAEN guideline 2014 suggests using fentanyl (1-2 g/kg) infused with paracetamol (1000 mg). This guideline does not recommend using morphine. Furthermore, NAEN guideline indicates that using ketamine (25 mg/kg IV) infused with midazolam (1mg, IV) and paracetamol (1000 mg IV) (10) is preferable in cases with evidence of hypovolemia or insecure airways (10). Administration of ketamine is recommended in four other protocols (11, 15, 16, 20) (Table 2). 

Intravenous paracetamol (two guidelines), nitric oxide (three guidelines), ibuprofen (three guidelines), ketorolac (two guidelines), methoxyflurane (three guidelines), non-steroidal anti-inflammatory drugs (one guideline) and diamorphine (one guideline) are alternative options in pre-hospital management of severe pain in adults. One guideline suggested prescription of codeine and tramadol while another one recommended aspirin prescription (Table 2). 

**Pain management in children (under 14 years)**

As mentioned, pre-hospital pain management in children was reported in 10 of the included studies. In these guidelines, different medications were proposed, which included morphine, fentanyl, ketamine, paracetamol, midazolam, nitric oxide, ketorolac, ibuprofen, methoxyflurane, codeine, tramadol and hydromorphone. In these guidelines, instructions for using medications were different according to the severity of pain.

***Pre-hospital management of mild pain in children***

Four guidelines precisely proposed instructions on pre-hospital management of mild pain in children. According to the guidelines, control and management of pain in a child who is having mild pain include appeasing the child’s pain or at most prescribing paracetamol in a dose of 10-20mg/kg and ibuprofen in a dose of 4-10 mg/kg (Table 3).

***Pre-hospital management of moderate pain in children***

Eight guidelines provided advice on pre-hospital management of moderate pain. Different medications are recommended for controlling and managing moderate pain in children. Six of these guidelines suggest using intravenous (IV), intranasal (IN) or intra-osseous (IO) fentanyl. Also, five guidelines proposed using paracetamol with a 15 mg/kg dosage, and four guidelines referred to morphine as a treatment option (Table 3). It is worth mentioning that only one guideline recommends intranasal ketamine in 0.5 mg/kg dosage for managing moderate pain (20).

Other recommended drugs in moderate pain management in children include methoxyflurane (three guidelines), ketorolac (two guidelines), nitric oxide (two guidelines), ibuprofen (two guidelines), and codeine and tramadol (one guideline) (Table 3).

***Pre-hospital management of severe pain in children***

10 guidelines provided instructions on pre-hospital management of severe pain in children. The first line of treatment in pre-hospital management of severe pain in children is prescribing morphine and fentanyl.

Nine guidelines suggested using fentanyl (1-2 μg/kg IV/IN/IO or 25-50 μg IN only) for severe pain management in children. Also, eight guidelines recommended morphine (0.05-0.1 mg/kg) as a treatment option. Three guidelines proposed intravenous, intranasal or intra-osseous ketamine as another option for pain management. Furthermore, a guideline suggested using intravenous ketamine (0.25 mg/kg) only when there is a severe trauma pain in the limbs, which is not responding to opioids (16).

Paracetamol (one protocol), nitric oxide (one protocol only after consultation), ketorolac (two protocols), methoxyflurane (two protocols) and hydromorphone (one protocol) are other drugs recommended for strict pain management in children (Table 3).

**Quality Control of Guidelines**

The overall score of the included guidelines varied from 4.5 to 6.5. Aim and scope domain rating of the guidelines varied from 88% to 100%, stakeholder involvement domain varied from 72% to 100%, rigor of development domain varied from 12% to 81%, applicability domain varied from 79% to 100% and editorial independence varied from 8% to 50%. In the domain of clarity of presentation, the score of all studies was 100% (Table 4).

## Discussion

The current systematic review summarized the existing evidence on pre-hospital pain management, evaluating available guidelines and protocols. These guidelines provided instructions based on age groups (adults and children) and pain severity. The majority of guidelines recommended paracetamol as the medication of choice for management of mild pain in adults and children. For management of moderate to severe pain in pre-hospital setting, fentanyl and morphine are the first line choices. Fentanyl is the first line treatment for children.

Although, some of the guidelines referred to ketamine as an alternative treatment for pain management, it seems that ketamine is still not the first line treatment in these conditions. However, it appears that ketamine should be prescribed instead of fentanyl if the patient is hypovolemic or the airway is not secure. Overall, there is no strong evidence supporting the usage of ketamine in pain management.

Only two guidelines reported levels of evidence in detail. All of the other guidelines depicted a description regarding how the guideline was achieved and levels of evidence. Indeed, levels of evidence presented in each guideline varied from the others. In general, levels of evidence presented for using fentanyl and morphine in pain management is moderate to high. In case of using ketamine, one guideline has referred to reported evidence as poor, and the other articles disregarded the usage of ketamine.

Overall, management of mild pain was mentioned as opioid-free in guidelines, while almost all the guidelines (except for two protocols in moderate pain management section) referred to fentanyl or morphine as the first line treatments for moderate to severe pain management. The two mentioned guidelines regarding management of moderate pain (15, 18) did not recommend using fentanyl and morphine and suggested using paracetamol with 1000 mg dosage instead. 

The majority of the guidelines provided single-drug protocols. Only in special circumstances, such as shock, hypovolemia and unsecure airways, multidrug protocols are suggested. In this regard, the NAEN, 2014 guideline recommended that if a patient is hypovolemic or has no secure airways, ketamine in 0.25 mg/kg dosage with midazolam in 1 mg dosage should be administered.

## Conclusion

The present systematic review has summarized the current evidence in pre-hospital pain management for the first time via investigation of guidelines and protocols concerning the matter. These guidelines presented instructions in age (adults and children) and pain severity categories. Based on the obtained evidence, most of the guidelines recommend paracetamol as the treatment of choice for mild pain in both children and adults. For moderate and severe pain management, fentanyl and morphine are suggested medications for both adults and children, between these two medications, fentanyl is the treatment of choice for children. In conclusion, opioid-free protocols still have no place in pre-hospital management of moderate to severe pain.

## Funding Support

This research has been supported by Tehran Medical Service Center grant.
